# Posterior Cruciate Ligament Reconstruction Using a Trans-septal Approach With Preserved Hamstrings Tibial Insertion

**DOI:** 10.1016/j.eats.2024.103278

**Published:** 2024-10-24

**Authors:** Paul Ruterana, Pierre Sautet, Victor Housset, Thomas Bauer, Bertrand Sonnery-Cottet, Charles Pioger

**Affiliations:** a Department of Orthopaedic Surgery, Ambroise Paré Hospital, Boulogne-Billancourt, France; b Centre Orthopedique Santy, Lyon, France

## Abstract

Posterior cruciate ligament reconstruction is a challenging procedure and requires a safe approach that provides a proper visualization of the tibial footprint while avoiding neurovascular damage and facilitating graft passage. Recent studies have shown that the preservation of the hamstring tibial attachment improves fixation and vascularization in the setting of anterior cruciate ligament reconstruction. This Technical Note reports an effective and reproducible arthroscopic single-bundle posterior cruciate ligament reconstruction through a trans-septal approach wit preserved hamstring tibial insertion.

Due to its rare indication, posterior cruciate ligament (PCL) reconstruction (PCLR) is a challenging procedure, and its effectiveness can be compromised by many technical issues. Untreated associated posterolateral injuries, improper graft tunnel placement, “killer turn” erosion, varus malalignment, and primary suture repair account for the main reasons for persistent instability.^1^ Residual posterior laxity can also be imputed to improper tibial fixation, and secondary attachment could mitigate this risk.^2^ Preservation of the hamstring tibial attachment is a widely employed method in anterior cruciate ligament (ACL) reconstruction. The tibial insertion is preserved to improve fixation and vascularity,^3^^,^^4^ which brings immediate mechanical stability and promotes graft remodeling.^5^ Also, PCLR requires a safe approach that provides a proper visualization of the tibial footprint while avoiding neurovascular damage and facilitating graft passage. We introduce here a single-bundle PCLR through a trans-septal approach with preserved hamstring tibial insertion (Video 1).

## Surgical Technique

The patient is placed in a supine position with a first lateral support at the level of the tourniquet and a second one under the foot to position the lower limb at 90° of knee flexion (Tables 1 and 2).

**Table 1:** Pearls and Pitfalls

Pearls
• Trans-septal approach facilitates suture-passing manipulation.
• Pushing as much of the passing suture loop as possible behind the medial plateau will make it easier to retrieve it from the tibial tunnel.
• Reconstruction length from tibial insertion to the distal mark is usually around 15 cm.
• Make sure to use a long suture loop to be able to report and measure the reconstruction length adequately.
Pitfalls
• Fluoroscopic control is recommended for first cases.
• If the posterolateral portal is made too posteriorly, there is a risk of injuring the common peroneal nerve.
• Direct creation of the trans-septal portal by the surgeon, involving posterior movement of the rod while disrupting the septum, may lead to neurovascular injury.

**Table 2:** Advantages and Disadvantages

Advantages
• No need for a canula.
• Better view of the tibial insertion of the PCL.
• Initially using a 6-mm reamer for the PCL femoral tunnel allows adjustments when using the larger reamer.
• Graft backup fixation and vascularization are improved.
• The technique is cost-effective (no additional specific implant is needed).
• The graft cannot fall on the floor.
Disadvantages
• Concomitant graft preparation and arthroscopic procedure are feasible but require a trained surgical assistant.
• Challenges in graft passage between the PCL remnant and adjacent soft tissues.
• Difficulty in determining exact tibial footprint of the PCL during the learning curve.

PCL, posterior cruciate ligament.

Video 1 describes the procedure on a patient’s right knee, and the following figures present the reconstruction on a left knee for photography quality purposes.

### Hamstring Harvest

The semitendinosus and gracilis tendons are harvested. Both tibial insertions are preserved. The tendons are wrapped in a gauze soaked in 2.5 mg/mL vancomycin solution (125 mg/50 mL) immediately after harvest for approximately 10 minutes^6^ and stored in the posterior thigh compartment.

### Exploration and Trans-septal Approach

After evaluation and treatment of associated lesions, including preparation for frequently associated posterolateral reconstruction, the preparation of the tibial tunnel can begin.

The knee is flexed to 90°, and the 30° arthroscope is pushed in the notch between the femoral medial condyle and the PCL. The transillumination allows the localization of the posteromedial (PM) portal safely with a needle before opening it with a scalpel blade. Then, the shaver can be introduced through the PM portal to remove the central inferior septum.

Once a breach is gently created, the scope is placed from the anteromedial portal to the posterolateral (PL) compartment to visualize the lateral aspect of the septum and to achieve complete removal of the septum.

The shaver is replaced by a switching stick to ease the camera insertion in the PM portal. A needle helps to localize the PL portal just behind the popliteus tendon, before opening it with a scalpel.

### Tunnels

**Fig 1:**
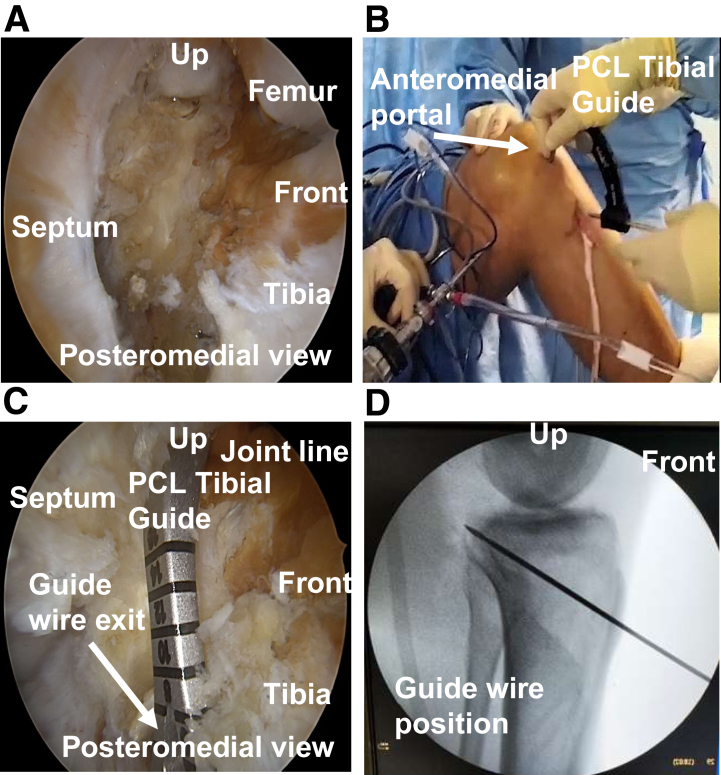
Posterior cruciate ligament (PCL) reconstruction of a left knee: tibial tunnel preparation under arthroscopic and fluoroscopic control. (A) Posteromedial view of the PCL footprint exposed. (B) Anterior view for PCL tibial guide positioning on a left knee. (C) Posteromedial view of PCL tibial guide positioning. (D) Fluoroscopic lateral view: guidewire position check.

Under direct visualization, the tibial PCL insertion is identified (Fig 1) and the remnant preserved if existing (Fig 1A). The extra-articular entry point of the specific PCL guide (Arthrex) is located at the proximal part of the hamstring harvesting incision, around 3 cm medial to the tibial crest (Fig 1B), and positioned under a direct PM arthroscopic view (Fig 1C). The final guidewire placement is controlled by fluoroscopy (Fig 1D).

Once the correct guidewire position is confirmed, the tunnel is carefully drilled with an 8- or 9-mm reamer with the protection of a curette introduced through the PL portal at the tip of the wire. Finally, soft tissues are removed using a shaver into the tibial tunnel to facilitate the graft passage.

The femoral tunnel is created in an inside-out freehand fashion. Under arthroscopic vision through the anteromedial portal, the drilling position is located 5 mm from the articular surface of the medial femoral condyle at an approximately 2-o’clock position on the right knee and a 10-o’clock position on the left knee. A 6-mm reamer is used first, followed by a 9-mm reamer, which allows to correct the direction. Another option is to create the femoral tunnel using an outside-in retro-socket technique. Soft tissue surrounding the tunnel entry is removed.

### Passing Suture

**Fig 2:**
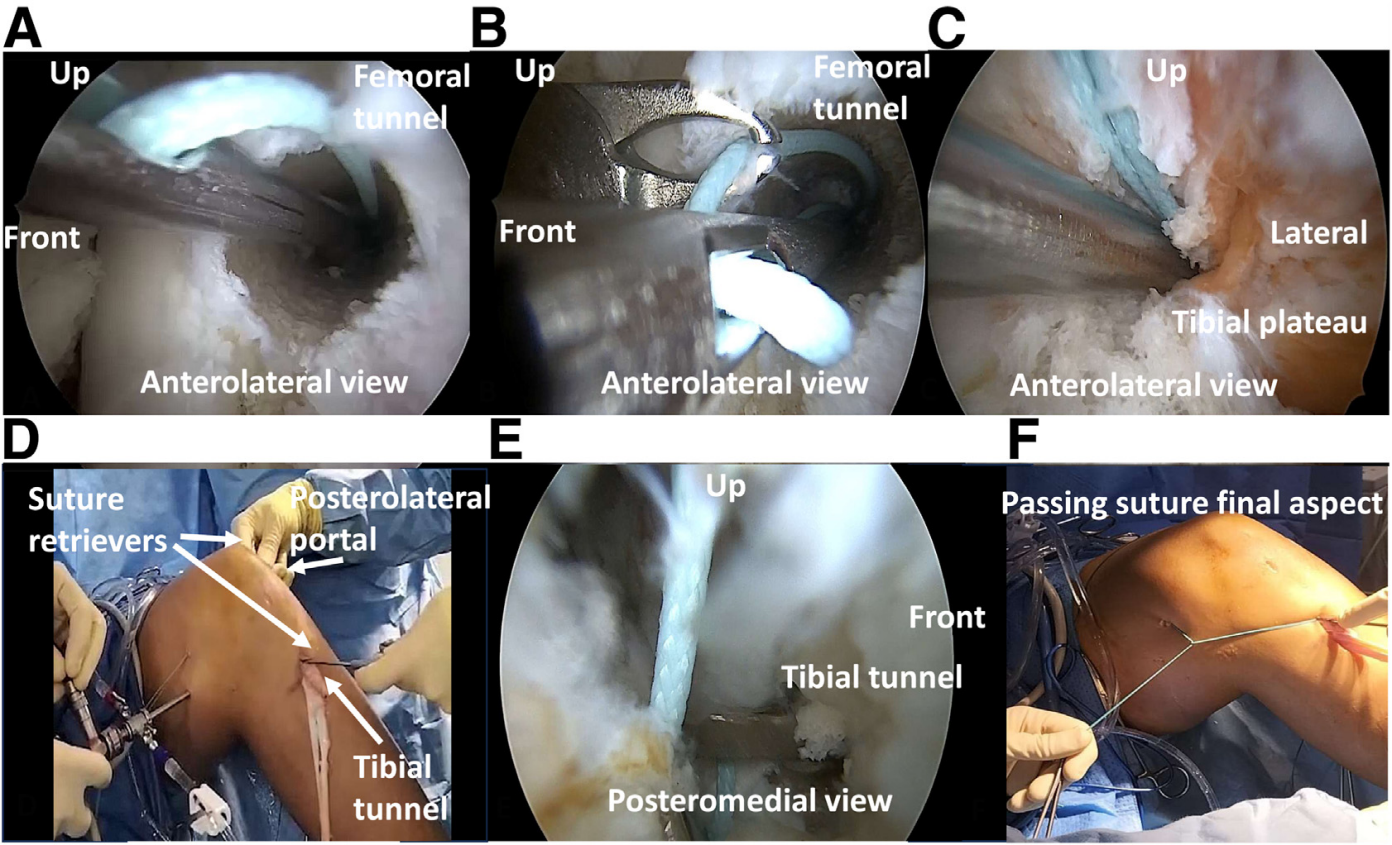
Posterior cruciate ligament reconstruction of a left knee: suture passing eased by the trans-septal approach. (A) Anterolateral (AL) view of the suture loop pushed down in the femoral tunnel. (B) AL view, loading of the loop in the suture retriever through the anteromedial portal. (C) AL view, suture loop pushed down behind the medial tibial plateau. (D, E) Scope switched to the posteromedial portal and loop helped in the tibial tunnel. (F) Suture loop final aspect before measures.

The surgeon forms a long downward loop with a thick braided suture (Mersuture No. 3; Ethicon). While the arthroscope is inserted in the anterolateral portal (Fig 2), the loop is pushed down in the femoral tunnel with an eyelet wire (Fig 2A). Then, through the anteromedial portal, it is loaded in a suture retriever (Fig 2B) and pushed down behind the medial plateau (Fig 2C). The scope is switched to the PM portal, and the loop is helped into the tibial tunnel with arthroscopic forceps (Fig 2 D and E). This passing suture (Fig 2F) will help to measure the length of graft needed.

### Measures

**Fig 3:**
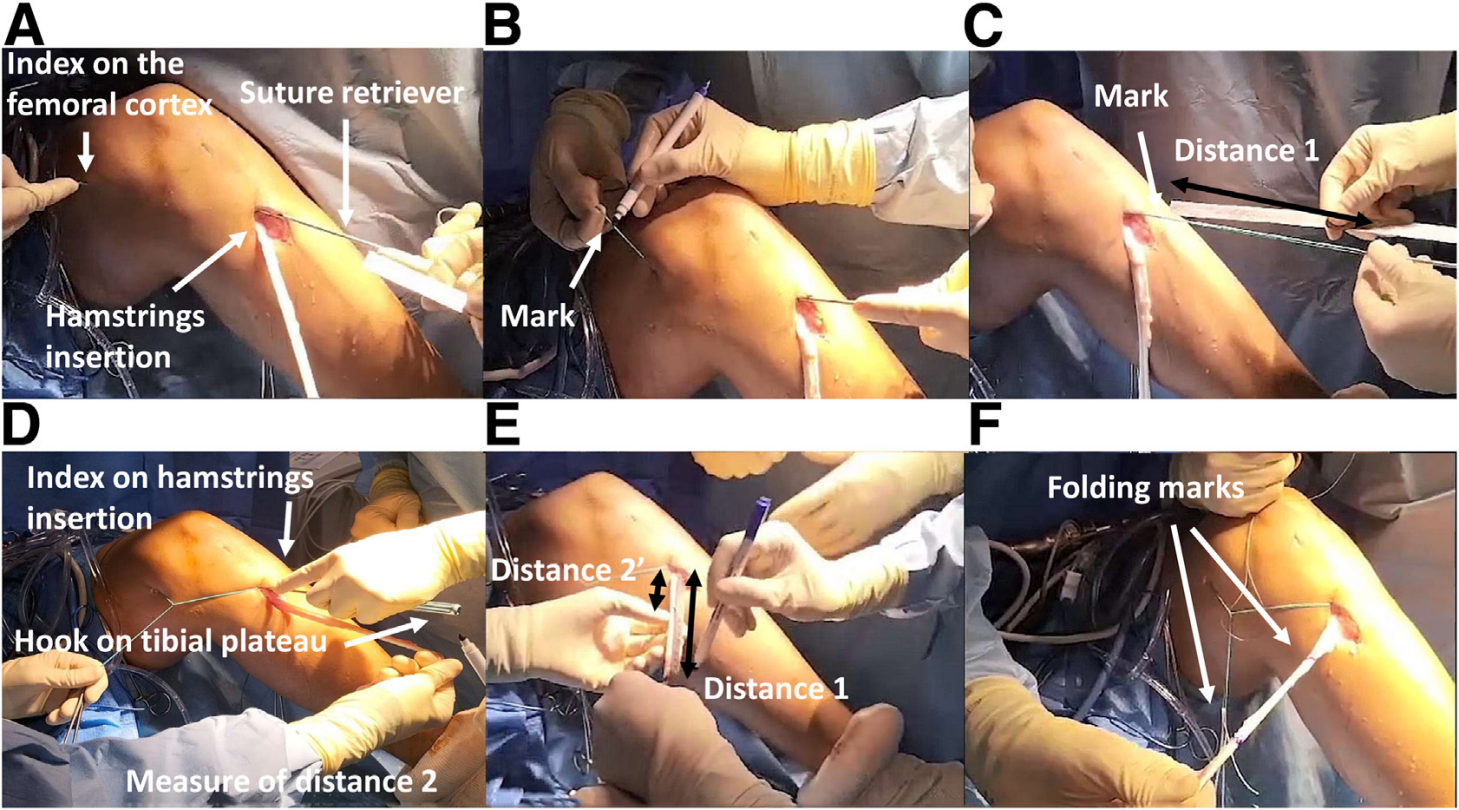
Posterior cruciate ligament reconstruction of a left knee: adequate graft length measure. (A) The loop is placed on the hamstring insertion. (B) The length between the hamstring insertion and the femoral cortex is plotted on the suture loop. (C) Measure of distance 1 between the loop end and the mark. (D) Measure of distance 2 from the tibial plateau to the hamstring insertion with the arthroscopic hook. (E) Starting at the hamstring insertion, distances 1 and 2 are plotted on the graft. (F) Final aspect of the graft with both marks.

The measuring system is greatly inspired by the combined ACL and anterolateral reconstruction described by Sonnery-Cottet et al. in 2016.^7^ The operator seizes the loop with arthroscopic forceps (Fig 3). The loop is placed on the hamstring insertion (Fig 3A). The operator grabs the proximal end of the passing suture as closely as possible to the femoral lateral cortex between their thumb and index finger and pulls the suture while accompanying the other side of the loop hooked in the arthroscopic forceps. The total length between the hamstring insertion and the femoral lateral cortex (distance 1) is plotted on the suture loop previously dried with a sterile dressing (Fig 3B). The surgeon pulls the suture loop outside to measure distance 1 between the loop end and the mark (Fig 3C). Distance 2 from the tibial plateau and hamstring insertion is measured with the arthroscopic hook, and 2 to 3 cm is subtracted from distance 2 to get 2 to 3 cm of doubled graft in the tibial tunnel (Fig 3D). Starting at the hamstring insertion (Fig 3E), distances 1 and 2 are indicated on the graft (Fig 3F).

### Graft Preparation

**Fig 4:**
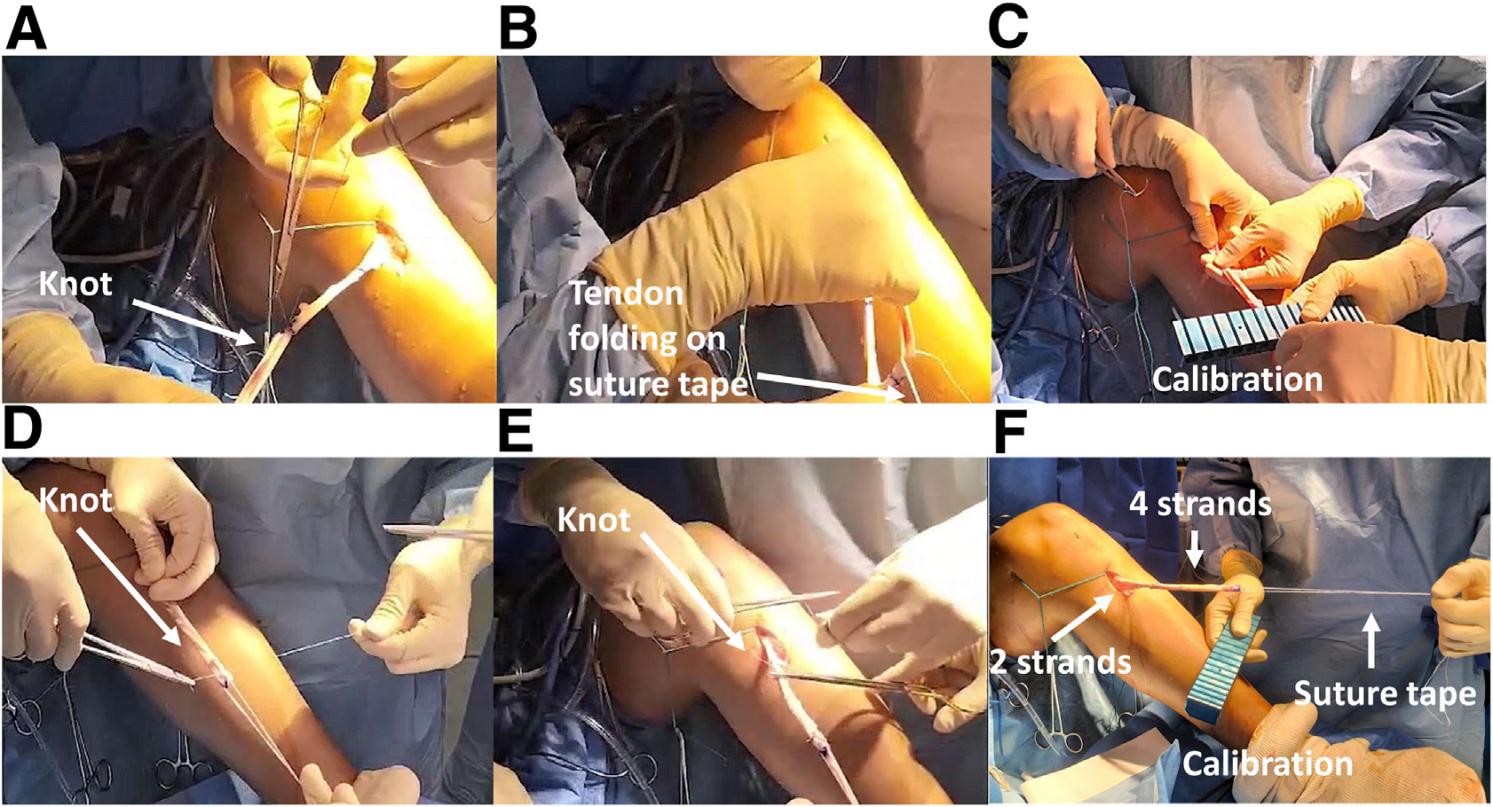
Posterior cruciate ligament reconstruction of a left knee: graft preparation and calibration to ensure tunnel passage. (A) A first knot is performed on the distal mark. (B) The tendons are folded 2 or 3 times on a flat braided suture tape at the distal mark. (C) Graft calibration before solidarization. (D) Strand union with 2 knots at both distal and proximal marks. (E) Definitive graft calibration.

A first knot with No. 0 Mersuture (Ethicon) is performed on the distal mark (Fig 4A). The tendons are then folded twice on a flat braided suture tape (Arthrex) at the distal mark (Fig 4B). It is important to calibrate the graft (Fig 4C) before solidarization by 2 knots of No. 0 Mersuture (Ethicon) at the distal (Fig 4D) and proximal (Fig 4E) marks. Definitive graft calibration is controlled (Fig 4F). To ease graft traction, a high-strength pulling suture (TigerWire; Arthrex) can be knotted at the distal end.

### Graft Passage and Fixation

**Fig 5:**
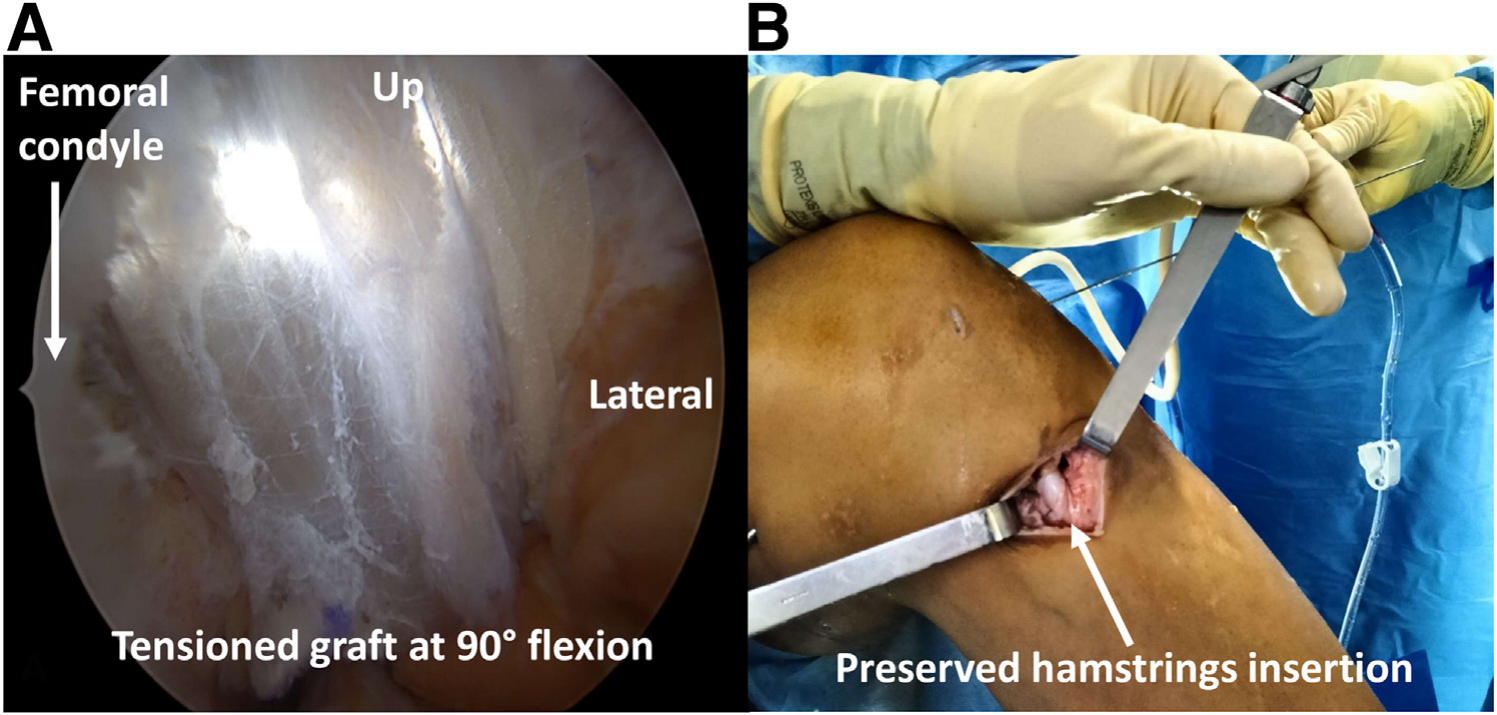
Posterior cruciate ligament reconstruction of a left knee: graft final aspect. (A, B) Intra- and extra-articular graft tension is controlled.

The hamstring tendon autograft is routed from the tibia to the femur. The “killer” turn is controlled with the arthroscope in the PM portal, and the graft can be helped with forceps in the posterolateral portal. It is then fixed on the tibial side with an interference screw. The PCL is then tensioned with the knee flexed to 90° while applying anterior translation to the tibia to reduce the posterior drawer without any risk of overcorrection^8^ (Fig 5).

### Postoperative Rehabilitation

The patient is allowed partial weightbearing immediately with a PCL dynamic knee brace, reducing the posterior drawer and limiting knee flexion at 90° during the first 6 weeks.

In this first phase, passive 0° to 90° motion and quadricipital rehabilitation are the main objectives of the 3 physiotherapy sessions per week.

## Discussion

PCLR is a demanding procedure that requires optimal visualization through a reproducible and safe technique. Persistent side-to-side posterior tibial translation can occur and tends to progressively exacerbate over time.^9^

Of all causes for residual laxity, misplacement of the tibial tunnel and insufficient tibial fixation are important risk factors.^10^ Therefore, it is mandatory to visualize the original PCL fibers at the tibial attachment site. The use of a trans-septal approach allows the surgeon to safely perform an accurate transtibial tunnel and spare the remnant fibers of the PCL as much as possible.^11^

During PCLR, the poor bone density of the posterior tibial plateau is a concern.^12^ To mitigate the risk of insufficient tibial fixation, the preservation of the hamstring tibial insertion appears to be an interesting option as a backup. In cases of ACL reconstruction, the preservation of the hamstring tibial insertion increases immediate mechanical stability by 65% in comparison with interference screw alone.^13^ Preservation of the hamstring insertion could also facilitate the ligamentization process of the graft.^4^

In 2023, Vari et al.^5^ confirmed these findings in a prospective study on 180 patients. Graft remodeling on a magnetic resonance imaging scan at 1 year was significantly higher when semitendinosus attachment was preserved during ACL reconstruction. The group with preserved semitendinosus attachment also showed a better functional score at 1 year. However, these results need to be confirmed in a randomized trial.

In summary, the use of a trans-septal approach and the preservation of hamstring tibial insertions in PCLR could offer a main advantage in providing immediate, solid, and physiological fixation of a multistranded graft, which could promote graft integration without technical complexity.

## Disclosures

All authors (P.R., P.S., V.H., T.B., B.S-C., C.P.) declare that they have no known competing financial interests or personal relationships that could have appeared to influence the work reported in this paper.

## Supplementary Data

Video 1 PCL reconstruction technique with the preservation of the hamstrings tibial insertion.
